# Antioxidant, Anti-acetylcholinesterase, Anti-inflammatory and DNA Protection Activities of *Glaucium grandiflorum v*ar.* grandiflorum*

**Published:** 2018

**Authors:** Nurten Ozsoy, Tugba Yilmaz-Ozden, Pinar Aksoy-Sagirli, Hasan Şahin, Aynur Sarı

**Affiliations:** a *Department of Biochemistry, Faculty of Pharmacy, Istanbul University, Istanbul, Turkey. *; b *Department of Pharmacognosy, Faculty of Pharmacy, Istanbul University, Istanbul, Turkey.*

**Keywords:** Glaucium grandiflorum var. grandiflorum, Antioxidant, Anti-cholinesterase, Anti-inflammatory, DNA protecting activity

## Abstract

In this study, antioxidant, antiacetylcholinesterase, anti-inflammatory, and DNA protecting activities of the aerial parts of *Glaucium grandiflorum *var. *grandiflorum *were evaluated. The lyophilized methanolic extract of the aerial parts of *G. grandiflorum *var. *grandiflorum* was investigated for potential *in-vitro* antioxidant properties in thiobarbituric acid test using the lipid peroxidation of liposomes, ferric ion reducing antioxidant power (FRAP), 1,1-diphenyl-2-picrylhydrazyl,2,2’-azinobis(3-ethylbenzothiazoline-6-sulphonate) free-radicals and hypochlorous acid scavenging assays. The extract demonstrated antioxidant activity in all the assays. The (AChE) inhibition capacity of the lyophilized methanolic extract at 320 μg/mL (80.75 ± 1.59%) was found to be strong and almost equal to the inhibition capacity of the positive control, galantamine (82.23 ± 2.21%) at 25 μg/mL. The significant AChE inhibitory activity suggests that the extract may be beneficial in the treatment of Alzheimer’s disease. The extract also showed inhibitory activity against plasmid DNA damage (94%), as well as COX-2 69.05%, which is a target for many anti-inflammatory and cancer-preventive drugs. These results indicate that *G. grandiflorum *var. *grandiflorum* methanolic extract is an excellent source of compounds with antioxidant, anti-acetylcholinesterase and anti-inflammatory properties that prevent DNA damage.

## Introduction

Alzheimer’s disease (AD) is a progressive degenerative neurologic disorder resulting in impaired memory and behavior. The inhibition of acetylcholinesterase (AChE) has been one of the most used strategies for the treatment of AD (1). Many plant-derived alkaloids that act as AChE inhibitors (AChE-I) are available for the symptomatic treatment of patients with mild to moderate AD and can be considered as models for the development of anti-Alzheimer drugs. However, there is still a need for the discovery of more effective compounds with lower toxicity and higher central nervous system penetration (2). Furthermore, compounds possessing not only the AChE inhibitory properties, but also anti-inflammatory or antioxidant properties, are considered attractive because of the possibility to create multitargeted drugs acting with different mechanisms all related to the disease (2). Plant families that have been considered as potential sources of alkaloids are Amaryllidaceae, Buxaceae, Apocynaceae, Papaveraceae, Lycopodiaceae, and Leguminosae (2). The genus *Glaucium* (Papaveraceae) is of interest because of its medicinally important alkaloids. The species of *Glaucium* have been used in traditional medicine as laxative, hypnotic, and antidiabetic agents. The leaves of the species of *Glaucium* are also used in the treatment of dermatitis (3). There have been previous studies on the different *Glaucium* species demonstrating effects of potential interest in AD therapy, including antioxidant, anti-inflammatory, and anti-cholinesterase activities (3- 6) 

*Glaucium grandiflorum *Boiss. & Huet is a perennial herb indigenous to various regions of the Middle East extending from the Eastern Mediterranean to Iran (7). In Turkey, *Glaucium grandiflorum *has two varieties as var. *grandiflorum* and var. *torquatum*, of which the latter is indigenous to Turkey (8). The fruits of *G. grandiflorum *have been used as folk medicine in Turkey for the purification of blood and in the treatment of opthalmic diseases. It is also reported that the sap of this plant have been used widely in Iran for the same purpose [9]. The therapeutic benefit of the plant is often attributed to its alkaloids.

Previous investigations on the alkaloids of* G. grandiflorum *revealed the existence of protoberberine, protopine, aporphine, and benzophenanthridine type alkaloids (7, 9-11). Prior findings on the alkaloids of Turkish samples of* G. grandiflorum *Boiss. & Huet var.* grandiflorum *collected from three different parts of Anatolia were as follows: Protoberberine ((-)-α-N- methylcanadine, (-)-β-N- methylcanadine), protopine (allocryptopine, protopine), aporphine (isocorydine, corytuberine, corydine), benzylisoquinoline ((+)-reticuline, and berbithine) type alkaloids (9, 11).

The reported anti-inflammatory and analgesic activities of the aerial parts of *G. grandiflorum *were related partly to the glaucine and tetrahydropalmatine alkaloids (3) 

The present study aimed to evaluate the possible antioxidant, anti-inflammatory, AChE inhibitory and DNA protecting activities of *G. grandiflorum *var.* grandiflorum *collected from Doruksaray in Erzincan. Phytochemical investigations have been carried out on this collection and allocryptopine, protopine have been isolated as major alkaloids. The minor alkaloids have been proven to be (-)-α-N- methylcanadine, (-)-β-N- methylcanadine, (+) -reticuline, and berbithine (9).

## Experimental

*Plant material and chemicals*

The aerial parts of *G. grandiflorum *var.* grandiflorum *were collected from Doruksaray in Erzincan (Turkey) in June 2013. Voucher specimens were deposited in the Herbarium of Istanbul University, Faculty of Pharmacy, Istanbul, Turkey (ISTE 101801).

For the assessment of antioxidant activity, soybean L-α-phosphatidylcholine Type IV-S,2,2-diphenyl-1-picrylhydrazyl(DPPH), α1-antitrypsin from human plasma, elastase from porcine pancreas, N-succinyl-ala-ala-ala-p-nitroanilide and NaOCl were purchased from Sigma-Aldrich (St. Louis, MO, USA); 2,2’-azino-bis(3-ethylbenzothiazoline-6-sulfonic acid) diammonium salt (ABTS), and rutin were purchased from Fluka (Buchs, Switzerland); L-ascorbic acid, 2,4,6-tripyridyl-S-triazine (TPTZ), thiobarbituric acid (TBA), trichloroacetic acid (TCA), iron (II) sulfate heptahydrate and ferric chloride were purchased from Merck (Darmstadt, Germany). 

For the assessment of AChE inhibitory activity, acetylthiocholine iodide (ATChI), AChE, 5,5′-dithiobis (2-nitrobenzoic acid) (DTNB) and galantamine hydrobromide were purchased from Sigma-Aldrich (St. Louis, MO, USA).

For the assessment of cyclooxygenase (COX)-2 inhibitory activity, enzyme immunoassay (EIA) kit (560131) and indomethacin were obtained from Cayman (Ann Arbor, MI, USA).

The DNA protecting activity was assessed using the pBR322 plasmid purchased from Thermo Scientific (Fisher Scientific, Pittsburgh, PA, USA).

*Preparation of the extract*

The aerial parts of the plant were dried in shade at room temperature then powdered using a blender. 20 g of dried and powdered aerial parts of the plant were extracted with 450 mL of MeOH 70% with continuous stirring for 3 days at room temperature. Finally, the extract was evaporated to dryness under reduced pressure at 40 ºC in a rotary evaporator. The crude extract was lyophilized (2 g) and kept at -20 °C until used. For the assessment of activities, the extracts were dissolved in metanol. All the analyses were performed using a microplate reader (Biotek, Winooski, VT, USA). 

*Determination of the total phenolic compounds*

Phenolic compounds in the extract of *G. grandiflorum* were estimated by a colorimetric assay, based on procedure described by Slinkard and Singleton (12). The results were expressed as mg gallic acid equivalents (GAE)/g of dry weight (DW). 

*Determination of the total flavonoid content*

Total flavonoid content was determined by using a colorimetric method described by Sakanaka *et al*. (13). The results were expressed as mg of (+)-catechin equivalents (CE) per g of DW. 

*Determination of the total antioxidant activity*

The antioxidant activity of the extract was compared to that of rutin, the most representive flavonol for phenolic plants.

*Inhibition of lipid peroxidation*

*(LPO).* LPO assay was based on the method described by Duh *et al.* (14). The formation of LPO products was assayed by the measurement of thiobarbituric acid reactive substances (TBARS) levels on the basis of malondialdehyde (MDA) reaction with TBA at 532 nm according to Buege and Aust (15). The percentage inhibition of LPO was calculated by comparing the results of the sample with those of controls not treated with the antioxidant using the following equation: 

Inhibition effect (%) = (1 - Absorbance of sample at 532 nm) x 100.

*Absorbance of control at 532 nm*

The DPPH^•^ scavenging activity of the extract was measured according to the procedure described by Brand-Williams *et al*. (16) and calculated by the following equation: 

DPPH^•^ scavenging activity (%) = (1 - Absorbance of sample at 517 nm/Absorbance of control at 517 nm) x 100. 

*Total radical-trapping antioxidant potential (TRAP) assay*.TRAP of the extract was measured using the trolox equivalent antioxidant capacity (TEAC) assay as described by Re *et al*. (17). The ability to scavenge ABTS radical cation (ABTS^•+^) was calculated by the following equation: 

ABTS^•+^ scavenging activity (%) = (1 - Absorbance of sample at 734 nm/Absorbance of control at 734 nm) x 100. 

*Ferric reducing antioxidant power (FRAP) assay.* The FRAP assay was carried out according to the procedure of Benzie and Strain (18). The standard curve was constructed using iron sulfate heptahydrate solution (0.125 – 2 mM), and the results were expressed as mM Fe ^2+^ equivalents. *Scavenging of hypochlorous acid (HOCl) (antitrypsin protection assay).* Reaction with hypochlorous acid was studied using elastase assay as described by Murcia *et al*. (19). HOCl was prepared immediately before use by adjusting NaOCl solution to pH 6.2 with dilute H_2_SO_4_, and its concentration was measured spectrophotometrically at 235 nm, assuming a molar extinction coefficient of 100. HOCl scavenging activity was quantified as inhibition of the inactivation of α1-antitrypsin by HOCl. α1-Antitrypsin activity was measured indirectly as elastase activity with N-succinyl-ala-ala-ala-p-nitroaniline as substrate, since elastase is inhibited by α1-antitrypsin (20). The results were expressed as percentage inhibition of elastase activity with respect to the reaction mixture without test compound (saline only). 

Inhibition (%) = (1 - Reaction rate of sample at 410 nm/ Reaction rate of control at 410 nm) x 100.

*Determination of AChE inhibitory activity*

The extract was screened for its AChE inhibitory activity through the modified Ellman’s spectrophotometric method (21). Galantamine hydrobromide was used as a standard.

Inhibition of AChE (%) = (1 - Reaction rate of sample at 412 nm/ Reaction rate of control at 412 nm) x 100. 

*COX-2 inhibitory activity*

The ability of the extract to inhibit recombinant human COX-2 was determined by calculating percent inhibition of prostaglandin production using an enzyme immunoassay (EIA) kit according to the manufacturer’s instructions (Cayman, USA). 

*DNA nicking assay*

DNA-protecting activity of the lyophilized methanolic extract of the aerial parts of *G. grandiflorum *var.* grandiflorum *from devastating effects of hydroxyl radicals generated by Fenton reagent was evaluated by DNA nicking assay described by Lee *et al.* (22). DNA nicking assay was performed using pBR322 plasmid DNA. The reaction mixture contained 0.5 μg plasmid DNA, Fenton’s reagent (30 mM H_2_O_2_, 50 µM ascorbic acid, and 80 µM FeCl_3_) followed by the addition of extracts and the final volume of the mixture was brought up to 20 μL using distilled water. The mixture was then incubated for 30 min at 37 °C. The DNA was analyzed on 1% agarose gel using ethidium bromide staining. Densitometric analysis was performed by using BIO1D software.

*Statistical analysis*

All measurements were made in triplicate. The results were evaluated using unpaired *t*-test with NCSS statistical computer package and expressed as mean ± standard deviation. Differences were considered significant at *p* < 0.05.

## Results and Discussion

Total extractable compounds, total phenolic and flavonoid contents of lyophilized methanolic extract obtained from the aerial parts of *G. grandiflorum *var.* grandiflorum *are shown in Table 1. The total phenolic content of the extract was found to be 3.54 ± 0.11 mg, in terms of GAE per gram of DW of the sample. The total flavonoid content of the extract was found to be 3.15 ± 0.14 mg, in terms of CE/g of DW of the sample. Previous investigations on phenolic compounds of *G. aleppicum* (6) revealed significantly higher levels of total polyphenols (18.8 GAE mg/g DW) compared to that of *G. grandiflorum *var.* grandiflorum*. Since earlier studies revealed that one of the more prominent properties of the flavonoids is their excellent radical scavenging ability (23), it has been proposed that the active constituents contributing to the antioxidant activity of the extract are the polyphenolic substances, particularly flavonoids.

**Table 1:** Total extractable compounds (EC), total phenolic compounds (PC) (as gallic acid equivalents) and total flavonoids (as catechin equivalents) in the lyophilized methanol extract from the aerial parts of *G. grandiflorum *var.* grandiflorum*

**Extract**	**EC** **(g/g DW)**	**PC** **(mg/g DW)**	**Flavonoid** **(mg/g DW)**
*G. grandiflorum *var.* grandiflorum *	0.1	3.54 0.11	3.15 0.14

Values were the means of three replicates   standard deviation

**Table 2.** Antioxidant activities of the lyophilized methanolic extract from the aerial parts of *G. grandiflorum *var. *grandiflorum*

**Extract**	**EC**_50_ ** (mg/mL)** A			**TEAC value** B **(mM)**	**FRAP value** C **(mM Fe** ^2+^ **)**
	**LPO inhibition**	**DPPH**	**ABTS**		
*G. grandiflorum*	^2.62 0.08a^	^2.03 0.17a^	^4.80 0.14a^	^2.11 0.015^ ^a^	^2.453 0.07^ ^a^
Rutin	^0.76 0.03b^	^0.150 0.07b^	^0.58 0.02b^	^2.11 0.005^ ^a^	^2.53 0.01^ ^a^

Values were the means of three replicates standard deviation. Values with different letters in the same column

were significantly (*p* 0.05) different.

A EC_50_ value: The effective concentration at which the LPO inhibitory activity was 50 %; DPPH and ABTS radicals were scavenged by 50 %.

B Expressed as mM Trolox equivalents

C Expressed as mM ferrous ions equivalents

Determined at 10 mg/mL

Determined at 1.25 mg/mL

**Table 3:** AChE inhibitory activity of the lyophilized methanolic extract from the aerial parts of *G. grandiflorum* var. *grandiflorum*

**Extract**	**AChE inhibitory activity (%)**			**EC**_50_ ** (mg/mL)**
	**0.32 mg/Ml**	**0.16 mg/mL**	**0.08 mg/mL**	
*G. grandiflorum *var. *grandiflorum*	80.75 1.59	59.97 0.39	43.22 1.97	0.12 0.01
	**25 μg/mL**	**12.5 μg/mL**	**6.25 μg/mL**	**EC**_50_ ** (μg/mL)**
Galantamine	82.23 2.21	71.02 3.56	47.94 1.40	7.07 0.5

Values were the means of three replicates standard deviation.

**Table 4:** Hypochlorous acid scavenging activity of the lyophilized methanolic extract from the aerial parts of *G. grandiflorum *var. *grandiflorum*, determined in antitrypsin protection assay

**Extract**	**Elastase inhibitory activity (%)**			
	**10 mg/mL**	**5 mg/mL**	**2.5 mg/mL**	**EC**_50_ ** (mg/mL)**
*G. grandiflorum *var. *grandiflorum*	92.73 1.4	45.06 1.4	10.50 2.5	5.87 0.07
	**0.625 mg/mL**	**0.31 mg/mL**	**0.16 mg/mL**	**EC**_50_ ** (mg/mL)**
Vitamin C	83.01 2.33	56.23 1.75	17.93 0.71	0.35 0.019

Values were the means of three replicates standard deviation.

**Table 5:** COX-2 inhibitory activity of the lyophilized methanolic extract from the aerial parts of *G. grandiflorum *var. *grandiflorum*

**Extract**	**COX-2 inhibitory activity (%)**			
	**10 mg/mL**	**5 mg/mL**	**2.5 mg/mL**	**EC**_50_ ** (mg/mL)**
*G. grandiflorum * *var. grandiflorum*	72.39 2.9	46.87 1.6	19.21 0.9	6.39 0.1
	**40 g/mL**	**20 g/mL**	**10 g/mL**	**EC**_50_ ** (g/mL)**
Indomethacin	92.13 1.3	58.66 1.1	34.41 0.6	17.11 0.41

Values were the means of three replicates standard deviation.

**Figure 1.**
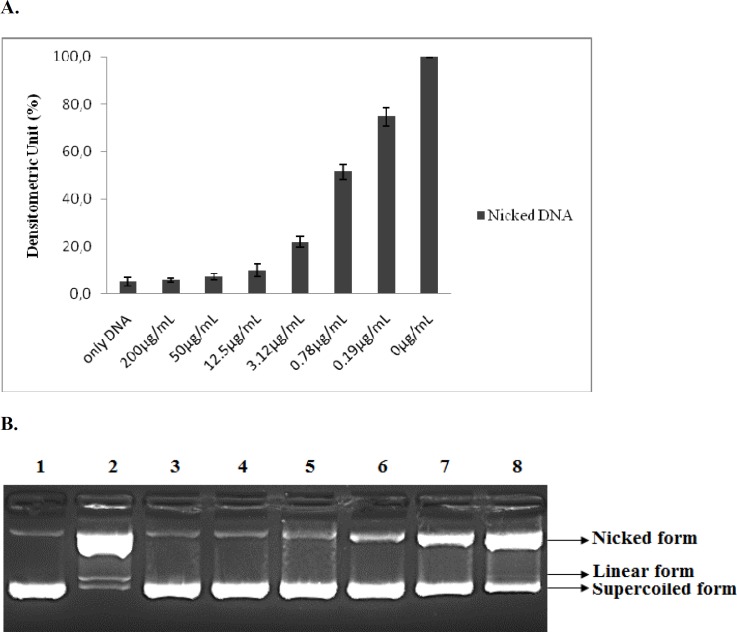
Protective effect of the lyophilized methanolic extract of *G. grandiflorum *var. *grandiflorum* in DNA nicking caused by hydroxyl radical. (A) The density of the nicked DNA form. Data are presented as mean ±SD (n=3). **(**B) Lane 1. Control (Distilled water + DNA), Lane 2. Negative control (DNA + Fenton's Reagent), Lane 3. *G. grandiflorum *var. *grandiflorum *(200μg/mL) + DNA + FR, Lane 4. *G. grandiflorum *var. *grandiflorum* (50 μg/mL) + DNA + FR, Lane 5. *G. grandiflorum *var. *grandiflorum* (12.5 μg/mL) + DNA + FR, Lane 6. *G. grandiflorum* var. *grandiflorum* (3.12 μg/mL) + DNA + FR, Lane 7. *G. grandiflorum *var. *grandiflorum* (0.78 μg/mL) + DNA + FR, Lane 8. *G. grandiflorum* var. *grandiflorum* (0.19 μg/mL) +DNA+FR

Antioxidant activities of the extract were expressed as half maximal effective concentration (EC_50_), TEAC, and FRAP values and presented in Table 2.

A well-recognized result of oxidant injury is peroxidation of membrane lipids to organic peroxyl radicals which initiates a chain reaction that may explain many membrane-mediated effects of reactive oxygen species (ROS). At 5 mg/mL the extract showed 75.85 ± 1.44% inhibitory effect on LPO. Similarly, the methanol extracts of *G.contortuplicatum*, *G. elegans *and *G. fimbrilligerum *were reported to show 55.18 ± 2.41%, 61.76 ± 1.23% and 89.16 ± 8.01%, respectively, linoleic acid peroxidation inhibition, using 40 μg of plant extract in the reaction mixture (4). Based on the EC_50_ values, the anti-LPO activity of the extract was significantly lower (*p* < 0.05) with an EC_50_ value of 2.62 ± 0.08 mg/mL in comparison with rutin (0.76 ± 0.03 mg/mL). Souri *et al.* (4) reported the higher inhibitory activity of lyophilized methanolic extracts of *G. contortuplicatum*, *G. elegans *and *G. fimbrilligerum *with an average IC_50_ value of 8.68 ± 2.63, 14.20 ± 0.61 and 4.19 0.78 µg/mL, respectively.

The extract showed appreciable DPPH scavenging activity (EC_50 _= 2.03 ± 0.17 mg/mL), but this activity was considerably (*p *< 0.05) lower than that obtained with the reference antioxidant, rutin (EC_50 _= 0.150 ± 0.07 mg/mL).

The extract exhibited ABTS^•+ ^scavenging activity of 2.11 ± 0.015 mM Trolox equivalents or 21.1 mM of Trolox per gram of DW at 10 mg/mL, was nearly equal to that of rutin (2.11 ± 0.005 mM) at 1.25 mg/mL and may be attributed to its electron-transfer abilities. Based on the EC_50_ values, ABTS^•+^ scavenging activity of the extract was significantly lower (*p* < 0.05) with an EC_50_ value of 4.80 ± 0.14 mg/mL in comparison with rutin (0.58 ± 0.02 mg/mL). In comparison to the methanolic extract obtained from *G. aleppicum* which showed 81.2 µM TEAC/g DW (6), it was evident that *G. grandiflorum *var. *grandiflorum* methanol extract is stronger antioxidant.

The FRAP assay measures the ability of the extracts to reduce TPTZ-Fe (III) complex to TPTZ-Fe (II) complex. It was found that at 10 mg/mL the lyophilized methanolic extract from *G. grandiflorum *var. *grandiflorum* possesses high reducing power (FRAP value = 2.45 ± 0.07 mM Fe^2+^) similar to that of rutin at 1.25 mg/mL (FRAP value = 2.53 ± 0.01 mM Fe^2+^).

The plant kingdom, an important source of several drugs or “lead compounds” for medicinal chemistry, is still largely unexplored despite the relatively large amount of tested plant extracts for AChE-I (2).

Since *Glaucium* species was found to be rich in alkaloids as active constituents that significantly contribute to their neuroprotective properties, the AChE inhibitory activity of the extract was also examined in this study. The extract showed the remarkable AChE inhibitory activity of 80.75 ± 1.59% at 0.32 mg/mL, which was comparable to that of galantamine (82.23 ± 2.21%) at a concentration of 25 μg/mL (Table 3). Compared with the data presented by Orhan *et al*. (5), the AChE inhibitory properties of the extracts of *G. grandiflorum var. grandiflorum *was similar to that of the chloroform:methanol (1:1); extract of the *G. corniculatum* (above 86.55 ± 0.67 inhibition rate at 1mg/mL). Based on the EC_50_ values, the AchE inhibitory activity of the extract was significantly lower (*p* < 0.05) with an EC_50_ value of 0.12 ± 0.01 mg/mL in comparison with galantamine (7.07 ± 0.5 µg/mL) (Table 3).

At chronic inflammation, activated neutrophils release H_2_O_2_ and the enzyme myeloperoxidase, catalyzing the formation of HOCl. It deactivates serine protease inhibitors such as α1-antitrypsin, α2-macroglobulin, plasminogen activator inhibitor, and tissue inhibitor of metalloproteinases 1 (20).

The mean elastase activity in the reaction mixture without test extract was 8.90 U/mL (A_410_ = 0.500). Elastase activity was inhibited 92.73% by the lyophilized methanolic extract of *G. grandiflorum *var. *grandiflorum *at 10 mg/mL (Table 4). Rutin showed no detectable HOCl scavenging activity. According to Murcia *et al.* (19) vitamin C is very good HOCl scavenger. Although the extract was less effective than the positive control, vitamin C, it is able to react with HOCl and thus to protect α1-antiproteinase against inactivation. Based on the EC_50_ values, the elastase inhibitory activity of the extract was significantly lower (*p* < 0.05) with an EC_50_ value of 5.87 0.07 mg/mL in comparison with vitamin C (0.35 ± 0.019 mg/mL) (Table 4).

Lyophilized methanolic extract of the aerial parts of *G.grandiflorum *var. *grandiflorum *was tested for its COX-2 inhibitory activity in comparison with indomethacin used as the positive control. COX-2, which is induced in response to inflammation, is important pharmacological target for the development of new drugs to treat chronic inflammatory diseases (24). The ability of the extracts to inhibit COX-2 was determined by calculating percent inhibition of prostaglandins production. At a concentration of 10 mg/mL, the extract showed inhibitory effect of 72.39%, but this activity was considerably (*p *< 0.05) lower than that of indomethacin. Based on the EC_50_ values, the COX-2 inhibitory activity of the extract was significantly lower (*p* < 0.05) with an EC_50_ value of 6.39 ± 0.1 mg/mL in comparison with indomethacin (17.11 ± 0.41 µg/mL) (Table 5).

The DNA nicking assay, which consisted of exposing a pBR322 plasmid DNA to the Fenton reaction, was designed to reveal increasing amounts of nicked and linear DNA in agarose gels. Addition of the lyophilized methanolic extract of *G. grandiflorum *var. *grandiflorum *resulted in a significant and dose dependent decrease in the formation of nicked DNA and it increased the supercoiled form of DNA. The plant extract showed maximum inhibition (94%) of plasmid DNA damage at 200 μg/mL, when the nicked form was normalized to the corresponding control treated with FR but no plant extract. The lowest extract dose tested (0.19 μg/mL) still inhibited DNA nicking by 25%. The concentration dependent protective activity of the plant extract is shown in 

Figure 1.

These results indicate that the *G. grandiflorum *var. *grandiflorum* extract is an excellent source of antioxidants that prevent DNA damage. To the best of our knowledge, no previous literature has reported the DNA protecting activity by *G. grandiflorum *var. *grandiflorum*. Therefore, the results reported here would appear to be the first report. 

## Conclusion

The antioxidative data presented in this study demonstrate that the lyophilized methanolic extract of *G. grandiflorum *var. *grandiflorum* is capable of scavenging some synthetic and naturally occurring free radicals, which indicates that the extract may have the potential to prevent free radical-mediated oxidative stress. Scavenging of HOCl and inhibition of COX-2 may contribute to the anti-inflammatory activities of the extract. The extract was also protective against DNA damage. Another important finding was the remarkable AChE inhibitory activity of the extract, which is of significant value in the pharmacological investigation for the treatment of AD. 
